# Fibronectin and laminin promote differentiation of human mesenchymal stem cells into insulin producing cells through activating Akt and ERK

**DOI:** 10.1186/1423-0127-17-56

**Published:** 2010-07-12

**Authors:** Hsiao-Yun Lin, Chih-Chien Tsai, Ling-Lan Chen, Shih-Hwa Chiou, Yng-Jiin Wang, Shih-Chieh Hung

**Affiliations:** 1 Stem Cell Laboratory, Department of Medical Research and Education, Veterans General Hospital-Taipei, Taiwan; 2 Institute of Biomedical Engineering, National Yang-Ming University, Taipei, Taiwan; 3 Institute of Clinical Medicine, National Yang-Ming University, Taipei, Taiwan; 4 Institute of Pharmacology, National Yang-Ming University, Taipei, Taiwan

## Abstract

**Background:**

Islet transplantation provides a promising cure for Type 1 diabetes; however it is limited by a shortage of pancreas donors. Bone marrow-derived multipotent mesenchymal stem cells (MSCs) offer renewable cells for generating insulin-producing cells (IPCs).

**Methods:**

We used a four-stage differentiation protocol, containing neuronal differentiation and IPC-conversion stages, and combined with pellet suspension culture to induce IPC differentiation.

**Results:**

Here, we report adding extracellular matrix proteins (ECM) such as fibronectin (FN) or laminin (LAM) enhances pancreatic differentiation with increases in insulin and Glut2 gene expressions, proinsulin and insulin protein levels, and insulin release in response to elevated glucose concentration. Adding FN or LAM induced activation of Akt and ERK. Blocking Akt or ERK by adding LY294002 (PI3K specific inhibitor), PD98059 (MEK specific inhibitor) or knocking down Akt or ERK failed to abrogate FN or LAM-induced enhancement of IPC differentiation. Only blocking both of Akt and ERK or knocking down Akt and ERK inhibited the enhancement of IPC differentiation by adding ECM.

**Conclusions:**

These data prove IPC differentiation by MSCs can be modulated by adding ECM, and these stimulatory effects were mediated through activation of Akt and ERK pathways.

## Background

Type 1 diabetes, caused by the autoimmune destruction of pancreatic β-cells, is deficient in insulin and requires exogenous insulin for treatment. Islet transplantation offers a potential cure for type 1 diabetes [1]. However, this approach is limited by a shortage of donor tissue suitable for transplantation. One alternative to islet transplantation is to implant a renewable source of insulin-producing cells (IPCs).

Stem cells have the potential to multiply and differentiate into any type of cells, thus providing cells that can generate IPCs for transplantation.

Human multipotent mesenchymal stem cells (MSCs) isolated from the bone marrow, can differentiate into multiple mesenchymal cell types, including cartilage, bone, and adipose tissues. They also display a neuronal phenotype after induction with growth factors, neurotrophic factors or chemical products like retinoic acid or 3-isobutyl-1-methylxanthine (IBMX) [2-5]. Although methods promoting neural differentiation have been adapted to derive IPCs from embryonic stem cells [6-9], such methods are insufficient to derive IPCs from MSCs [10]. For future application of MSCs, many efforts have been made to provide new protocols for differentiating MSCs into IPCs [10-12].

Interaction of extracellular matrix proteins (ECM) plays important roles in controlling cell proliferation, motility, cell death and differentiation of stem cells or progenitor cells. Pancreatic ECM mainly consists of fibronectin (FN) and laminin (LAM). Pancreatic FN is noted beneath the endothelial cells and epithelial ducts [13], while LAM is mainly present in basement membranes that form the interface between the epithelia and connective tissues [14]. Both FN and LAM affect β-cell differentiation, proliferation, and even their insulin secretion [15]. We have also demonstrated adding FN stimulated IPC differentiation by MSCs [10]; however, the molecular signaling pathways that ECM mediate to enhance IPC differentiation remain to be clarified.

Most of the MSCs used in previous studies were derived from primary cell cultures. Primary cells harvested from patients may have disease- or age-related differences such that results may be donor specific. We therefore chose to use an immortalized MSC line to provide more consistent results for parametric studies designed to optimize differentiation procedures. We also chose a four-stage differentiation protocol, containing neuronal differentiation and IPC-conversion stages, combined with pellet suspension culture for getting efficient IPC differentiation [10]. In our current study, we compared the effects of adding ECM such as FN and LAM on the expression of Insulin and Glucose transporter 2 (Glut2) genes and proinsulin and insulin protein levels. We further clarified the underlying mechanism that ECM mediated to enhance IPC differentiation and found this effect is mediated through activation of Akt and ERK.

## Methods

### Cell Lines and Culture Conditions

The human MSCs were established following retroviral transduction with the type 16 human papilloma virus proteins E6E7 and nucleoporation with human telomerase reverse transcriptase (hTERT) as previously described [3,16]. The cells were grown in a complete culture medium [CCM: DMEM-low glucose (LG) (Gibco, Grand Island, NY) supplemented with 10% fetal bovine serum (FBS), 100 U/mL penicillin, and 10 μg/mL streptomycin] at 37°C under 5% CO_2 _atmosphere. The medium was changed twice a week and subculture was performed at 1:5 split every week.

### IPC differentiation protocol

For IPC differentiation in pellet suspension culture, undifferentiated cells (stage 0) were suspended with CCM and aliquots of 2.5×10^5 ^cells were placed in 15 ml conical centrifuge tubes (Nalge Nunc International, Rochester, NY), centrifuged at 600 g for 5 min, and cultured in CCM for overnight. Then the pellets were lifted to float in the medium by patting the tube and the medium was replaced with CCM without (control) or with adding 5 μg/mL fibronectin (bovine plasma; F1141, Sigma) or laminin (basement membrane of Engelbreth-Holm-Swarm tumor; L2020, Sigma) for 2 days (stage I). At stage II, the pellets were switched into a medium prepared from 1:1 mixture of DMEM/F-12 medium containing 25 mM glucose (Invitrogen, Carlsbad, CA), Insulin-Transferin-Selenium-A (ITS-A, Sigma), and 0.45 mM isobutylmethylxanthine (IBMX; Sigma) without or with 5 μg/mL fibronectin or laminin for 1 day. Then the pellets were transferred into DMEM/F-12 medium containing 5.56 mM glucose, 10 mM nicotinamide (Sigma), N2 supplement (Invitrogen), and B27 supplement (Invitrogen) without or with 5 μg/mL fibronectin or laminin for 4 days (stage III). At stage IV, pellets were transferred into a medium with the same supplements at stage III but containing 25 mM glucose for 3 days. For identifying signaling pathways involved in IPC differentiation by MSCs, LY294002 (50 μM; Cell Signaling Technology) or/and PD98059 (50 μM; Cell Signaling Technology, Beverly, MA) were added from the start of stage III to the end of stage III.

### RT-PCR and quantitative RT-PCR

Total RNA was prepared by using the TRIzol^® ^Reagent (Invitrogen). For cDNA synthesis, random sequence primers were used to prime the reverse transcription reactions and synthesis was carried out by SuperScript™ III Reverse Transcriptase (Invitrogen). A total of 35 cycles of PCR were performed using Taq DNA polymerase Recombinant (Invitrogen). The reaction products were resolved by electrophoresis on a 1.2% agarose gel and visualized using ethidium bromide with the housekeeping gene (β-actin) as a control. For real-time PCR, the amplification was carried out in a total volume of 25 μL containing 0.5 μM of each primer, 4 mM MgCl_2_, 12.5 μL of LightCycler™-FastStart DNA Master SYBR green I (Roche Molecular Systems, Alameda, CA) and 10 μL of 1:20 diluted cDNA. PCR reactions were prepared in duplicate and heated to 95°C for 10 min followed by 40 cycles of denaturation at 95°C for 15 seconds, annealing at 60°C for 1 min, and extension at 72°C for 20 seconds. Standard curves (cycle threshold values versus template concentration) were prepared for each target gene and for the endogenous reference (GAPDH) in each sample. The quantification of the unknown samples was performed by the LightCycler Relative Quantification Software version 3.3 (Roche).

### Immunohistochemistry

Suspension cell pellets were fixed in 4% paraformaldehyde, then dehydrated and embedded in paraffin. Immunohistochemistry was performed on 4-μm tissue sections. The sections were first reacted with primary antibodies against human insulin (anti-insulin, 1:200; Chemicon, Temecula, CA) and proinsulin (anti-proinsulin, 1:200; Chemicon) followed by incubation with biotinylated secondary antibodies. Detection was accomplished using streptavidin-peroxidase conjugate and diaminobenzidine (DAB) as a substrate (LAB Vision, Fremont, CA). Counterstaining was carried out with hematoxylin. Finally, the slides were mounted and analyzed using an optical microscope.

### In Vitro Insulin Release Assay

Cell pellets after differentiation were rinsed twice in PBS and Krebs-Ringer bicarbonate (KRB) buffer (120 mM NaCl, 5 mM KCl, 2.5 mM CaCl_2_, 1.1 mM MgCl_2_, 25 mM NaHCO_3_, 0.1 g BSA) and preincubated for 1hour with KRB buffer containing 5 mM glucose. The pellets were then incubated for 1 hour in fresh KRB buffer with 5 mM, 10 mM, 15 mM or 25 mM glucose. Different agonists and antagonists of signal pathway of insulin release were added, including IBMX (100 μM) and nifedipine (50 μM) (Sigma). Insulin levels were measured using an enzyme-linked immunosorption assay (ELISA), which detects human insulin but not proinsulin or c-peptide.

### Cell Viability Assay

Cell viability was measured by 3-(4,5-dimethylthiazol-2-yl)-2,5-diphenyltetrazolium bromide (MTT) dye absorbance according to the manufacturer's instructions (Boehringer Mannheim, Mannheim, Germany). Cells were seeded in 96-well plates at a density of 10,000 per well. Cells were incubated without or with LY294002 (50 μM) or PD98059 (50 μM) for 48 hours. Cell viability was determined using MTT assay. Each experimental condition was done in triplicate and repeated at least once.

### Western Blotting

Cell lysates were prepared using protein extraction reagent (M-PER, Pierce, Illinois) plus protease inhibitor cocktail (Halt, Pierce). Protein concentrations were determined using the BCA assay (Pierce). After being heated for 5 min at 100°C in a sample buffer, aliquots of the cell lysates were run on a 10-12% SDS-polyacrylamide gel. Proteins were transferred to PVDF membrane. The membrane was blocked for more than 1 hour and then incubated overnight at 4 °C with the primary antibodies such as phosphate-ERK (Thr202/Tyr204) (Cell Signaling Technology), total-ERK (Cell Signaling Technology), phosphate-Akt (Cell Signaling Technology), total-Akt (Cell Signaling Technology) and Actin (Santa Cruz Biotechnology, Santa Cruz, CA). The membrane was washed and bound primary antibodies were detected by incubating at room temperature more than 1 hour with horseradish peroxidase-conjugated goat anti-rabbit IgG (Santa Cruz Biotechnology) and anti-goat IgG (Santa Cruz Biotechnology) for Actin. The membrane was washed and developed using a chemiluminescence assay (Perkin-Elmer Instruments, Inc. Boston, MA).

### Lentiviral-Mediated RNAi

The expression plasmids and the bacteria clone for Akt shRNA (TRCN0000010062) and ERK shRNA (TRCN0000010049) were provided by the National Science Council in Taiwan. Lentiviral production was done by transfection of 293T cells using Lipofectamine 2000 (LF2000; Invitrogen, Carlsbad, CA). Supernatants were collected 48 h after transfection and then were filtered. Subconfluent cells were infected with lentivirus in the presence of 8 μg/mL polybrene (Sigma-Aldrich). At 24 hours post-infection, we removed medium and replaced with fresh growth medium containing puromycin (1 μg/mL) and selected for infected cells for 48 hours.

## Results

### FN and LAM enhance differentiation of MSCs into insulin producing cells

**Figure 1:**
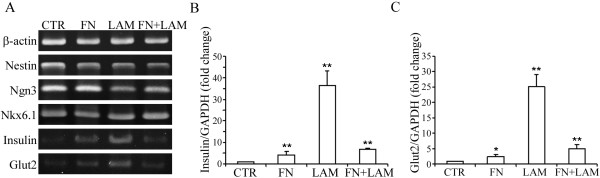
**Adding FN or LAM during differentiation enhances expression of insulin and Glut2**. MSCs were induced by four-stage protocol, and (A) RT-PCR and quantitative RT-PCR for (B) insulin and (C) Glut2 were performed at stage IV. Adding FN or LAM does not increase the expression of Nestin, Ngn3 and Nkx6.1, but increases the expression of Insulin and Glut2. (mean ± S.D.; **indicates significant difference (*P *< 0.01) compared with control by student's t test.)

To examine the effects of ECM, FN and LAM on IPC differentiation by MSCs, a four-stage differentiation protocol in suspension pellet culture [10] was performed and gene expression profiles for neural and pancreatic islet differentiation markers were assessed using RT-PCR for the stage IV cells. Nestin, the marker of neural precursor was expressed by MSCs both with and without FN and LAM. MSCs with or without these ECM also expressed genes specifying transcription factors essential for in vivo differentiation of IPCs, including Nkx6.1 and Ngn3 (Figure 1A). There was no obvious difference between the expression of these genes in cells treated with or without ECM. We then quantified the gene expression of insulin and Glut2. RT-PCR revealed adding FN or LAM increased the expression of Insulin and Glut2 (Figure 1A). Furthermore, quantitative RT-PCR showed adding FN and LAM increased the gene expression of insulin 5-fold and 52-fold, respectively (Figure 1B); and increased the gene expression of Glut2 4-fold and 29-fold, respectively (Figure 1C), compared to the cells without added ECM. However, combining FN and LAM did not further increase the expression of insulin and Glut2 (Figure 1), suggesting FN and LAM did not work synergistically to enhance IPC differentiation.

**Figure 2:**
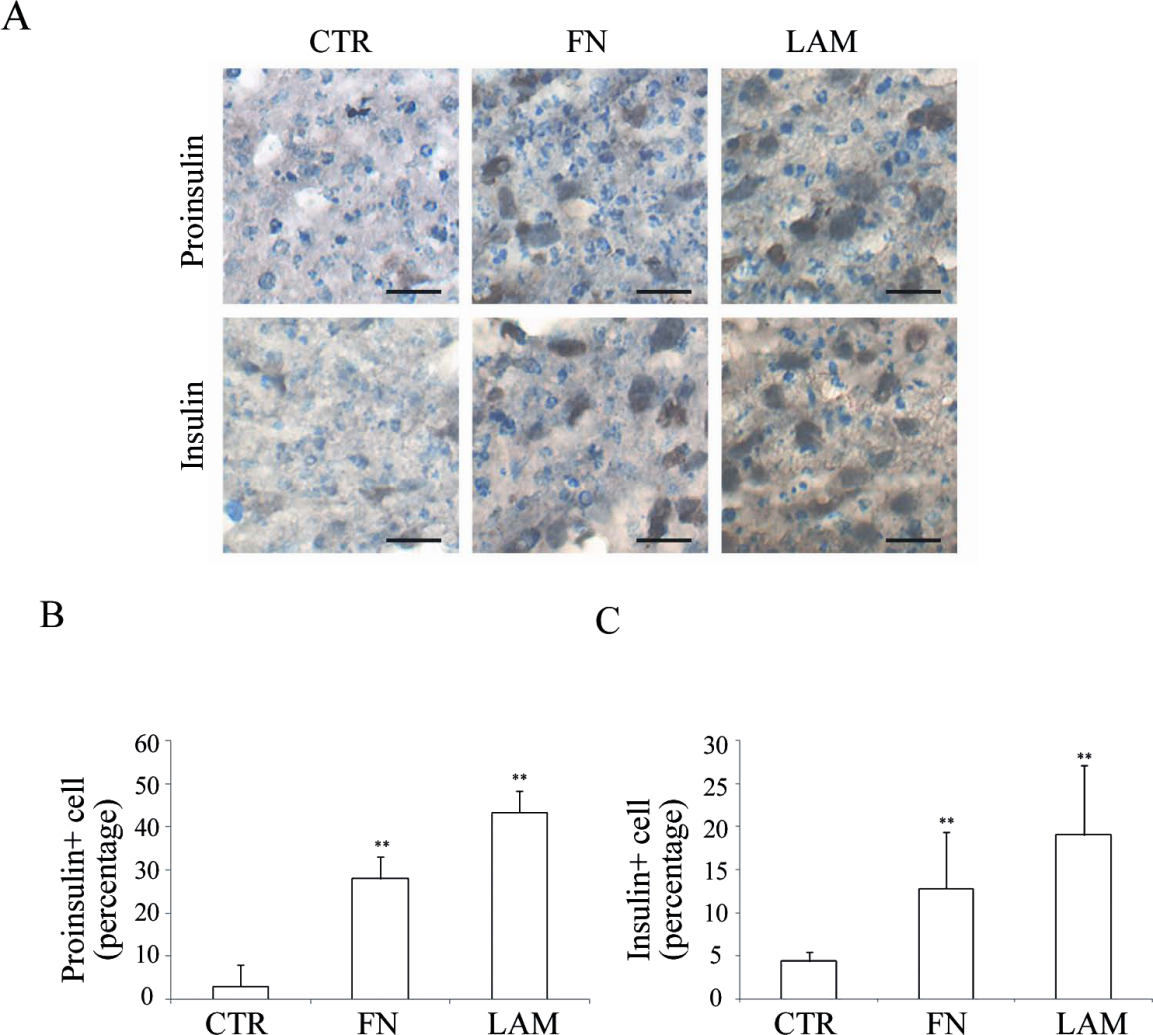
**Adding FN or LAM during differentiation enhances protein levels of proinsulin and insulin**. MSCs were induced by four-stage protocol, and immunohistochemistry was performed for stage IV cells. (A) Immunohistochemistry shows the expression of insulin and proinsulin in stage IV cells. Quantification of IHC staining shows FN and LAM increases the percentage of (B) proinsulin, and (C) insulin positive cells. (mean ± S.D.; ** indicates significant difference (*P *< 0.01) compared with control by student's t test.) (Bar = 100 μm).

Immunohistochemistry (IHC) in stage IV cells further revealed adding ECM increased the percentage of proinsulin and insulin expressing cells with the maximum effect seen in cells treated with LAM (Figure 2). These data are consistent with the mRNA levels of Insulin and Glut2 and all demonstrate LAM has greater ability than FN to stimulate IPC differentiation. These results indicate adding ECM, especially LAM, enhances differentiation of MSCs into IPCs.

### FN and LAM increases insulin release after glucose challenge

**Figure 3:**
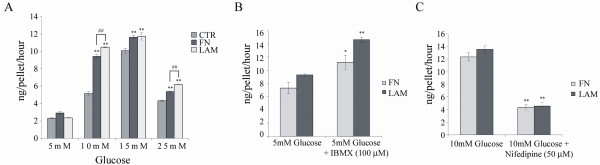
**Adding FN or LAM during differentiation increases insulin release in response to elevated glucose concentration**. MSCs were induced by four-stage protocol, and ELISA analysis for insulin release was performed for stage IV cells. (A) Insulin release at different glucose concentrations. Insulin release before and after treatment with (B) IBMX or (C) nifedipine. (mean ± S.D.; *P < 0.05 and **P < 0.01 compared with control by student's t test. ##*P *< 0.01 by student's t test.)

To quantify functional insulin release by stage IV cells, we used glucose-challenge test and assayed with a human insulin ELISA. A baseline release of insulin by stage IV cells was detected at 5 mM glucose, while the increase in glucose concentration to 10, 15 or 25 mM significantly increased insulin release with the greatest release at 15 mM (Figure 3A). These results suggest the release of insulin is dependent on extracellular glucose concentration. Both FN and LAM increased insulin release by stage IV cells at glucose concentrations of 10, 15 and 25 mM. The greatest difference of insulin release by cells treated with or without ECM was noted at 10 mM glucose, where FN and LAM increased insulin release roughly 1.8-fold and 2-fold, respectively, compared to cells without ECM. To determine if the cell pellets used physiological signaling pathways to regulate insulin release, we examined the effects of several agonists or antagonists on insulin release of ECM-induced cell pellets. Agonist- IBMX, an inhibitor of cyclic-AMP (cAMP) phosphodiesterase, stimulated insulin release in the presence of low glucose concentration (5 mM) (Figure 3B). Conversely, antagonist- nifedipine, a blocker of L-type Ca2 + channel (one of the Ca2 + channel present in β-cells), inhibited insulin release in the presence of 10 mM glucose concentration (Figure 3C). These results demonstrate stage IV cells secrete insulin in response to an increase in glucose concentration using the normal secreting mechanism of pancreatic islets.

### FN and LAM enhances activation of Akt and ERK

**Figure 4:**
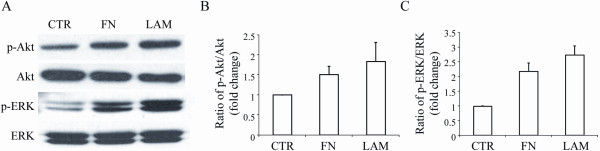
**Adding FN or LAM increases the activation of Akt and ERK**. (A) MSCs were induced for differentiation without (Control) or with FN or LAM, and western blotting was done for stage III cells. Quantification of western blotting shows FN or LAM increases the activation of (B) Akt and (C) Akt. (mean ± S.D.).

The ECM bind to cells by activating signaling molecules such as Akt and ERK. Therefore, we analyzed the effect of FN and LAM on the phosphorylation status of Akt and ERK for stage III cells. There was a baseline of Akt and ERK phosphorylation without adding ECM. FN and LAM increased phosphorylation of Akt and ERK, and LAM had greater effects on Akt and ERK activation than FN (Figure 4A). FN and LAM activated phosphorylation of AKT approximately 1.7-fold and 2.1-fold compared to the control, respectively (Figure 4B), and activated phosphorylation of ERK roughly 2.4-fold and 4-fold compared to the control, respectively (Figure 4C). These results showed both FN and LAM could enhance the phosphorylation of Akt and ERK.

### FN and LAM-induced enhancement of IPC differentiation depends on Akt and ERK activation

**Figure 5:**
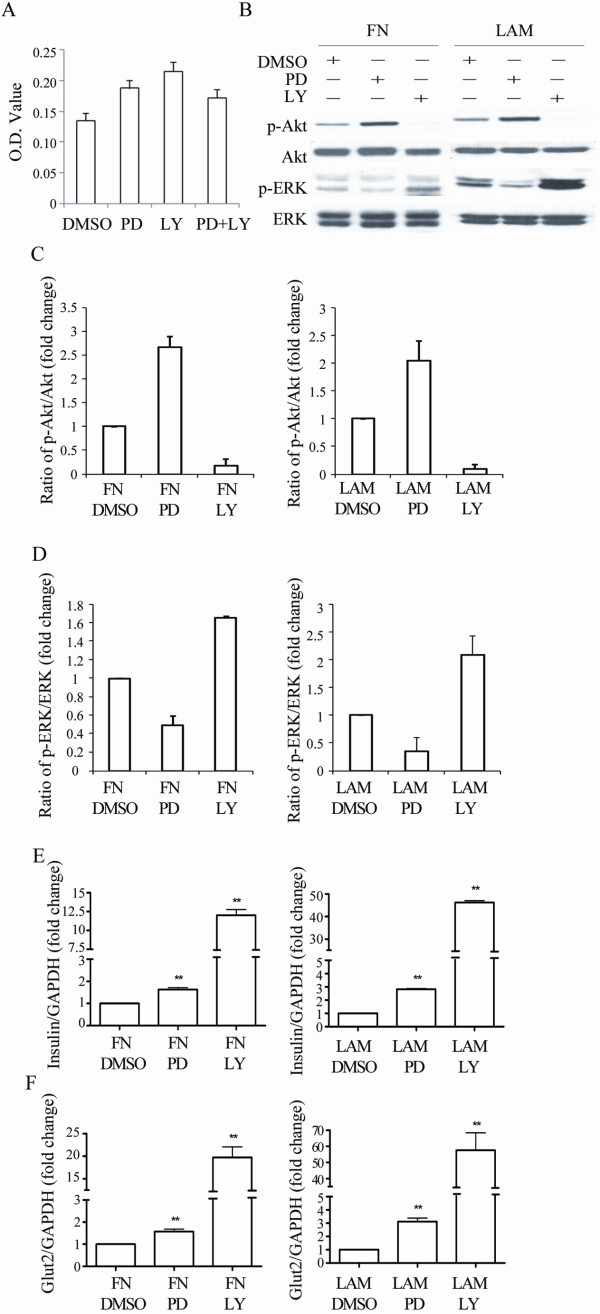
**Cross-talk between PI3K-Akt and MEK-ERK pathways during differentiation of MSCs into IPCs**. (A) MSCs were treated without (DMSO) or with 50 μM of PD98059 or LY294002 and MTT assay were performed at 48 hours. (B) MSCs were pretreated without (DMSO) or with PD98059 or LY294002 at stage III during differentiation with FN or LAM, and western blotting was done for stage IV cells. Quantification of Akt (C) and ERK (D) phosphorylation shows PD98059 and LY294002 increase the activation of Akt and ERK, respectively. Quantitative RT-PCR for (E) insulin and (F) Glut2 expression shows both PD98059 and LY294002 increase the expression of insulin and Glut2. (mean ± S.D.; **indicates significant difference (*P *< 0.01) compared with control by student's t test.).

**Figure 6:**
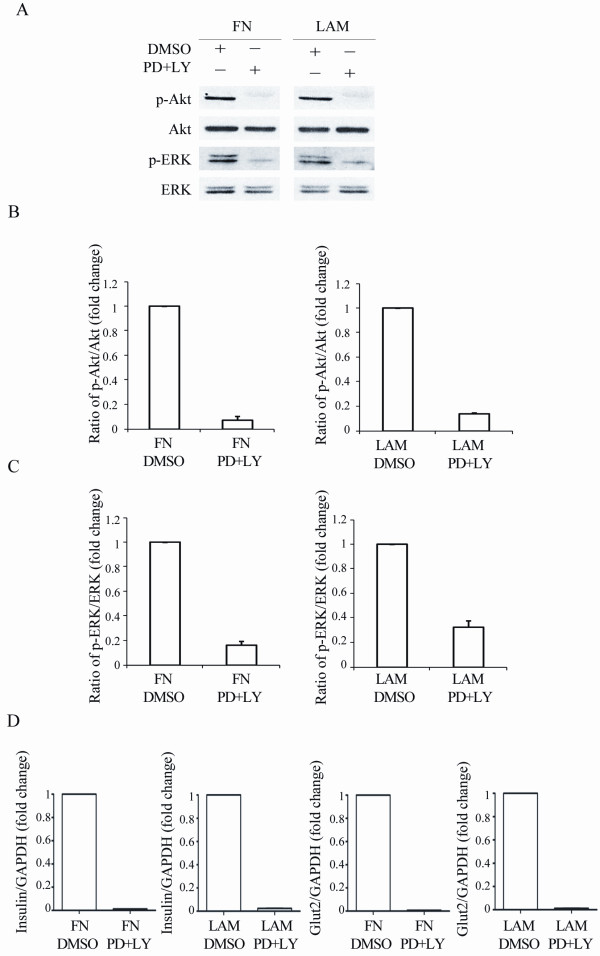
**FN or LAM enhances IPC differentiation by activating Akt and ERK**. (A) MSCs were pretreated without (DMSO) or with both PD98059 and LY294002 (PD+LY) at stage III during differentiation with FN or LAM, and western blotting was done for stage IV cells. Quantification of Akt (B) and ERK (C) phosphorylation shows adding both PD98059 and LY294002 decreases the activation of Akt and ERK. (D) Quantitative RT-PCR for insulin and Glut2 expression shows adding both PD98059 and LY294002 decreases the expression of insulin and Glut2. (mean ± S.D.).

**Figure 7:**
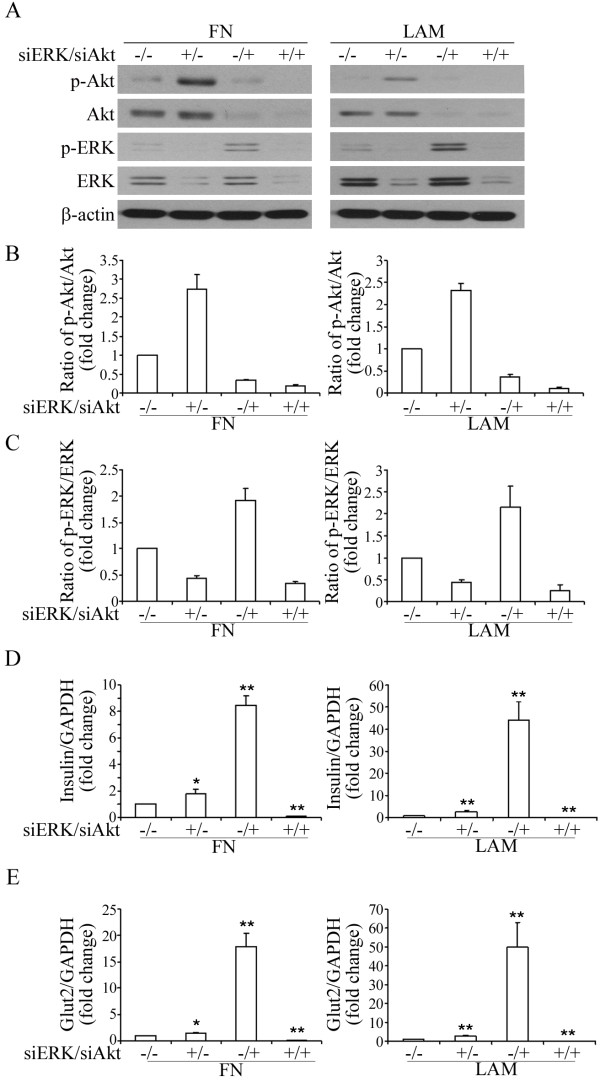
**The involvement of Akt and ERK activation in FN or LAM-induced enhancement of IPC differentiation**. (A) MSCs were transduced with scrambled (-) or siRNA against ERK (siERK) and Akt (siAkt) and induced for IPC differentiation with FN or LAM, and western blotting was done for stage IV cells. Quantification of Akt (B) and ERK (C) phosphorylation after transduction with siERK and siAkt. Quantitative RT-PCR for (D) insulin and (E) Glut2 expression shows transduction with either siERK or siAkt increases the expression of insulin and Glut2, and transduction with both siERK and siAkt decreases the expression of insulin and Glut2. (mean ± S.D.; *p < 0.05 and **p < 0.01 compared with the scrambled as determined by the student's t test.).

To examine the involvement of Akt and ERK activation in enhancing IPC differentiation by FN and LAM, the cells were pretreated with LY294002 (a specific inhibitor of PI3 Kinase) or PD98059 (a specific inhibitor of MEK 1) followed by induction with FN or LAM. Treatment with 50 μM of LY294002 or PD98059 did not induce any decrease in cell growth in MSCs (Figure 5A), suggesting these two reagents did not induce significant cytotoxicity. LY294002 decreased the activation of Akt both in cells treated with FN or LAM (Figure 5B and 5C). Surprisingly, we also found LY294002 increased the activation of ERK approximately 1.7-fold and 2.1-fold compared to FN and LAM treated controls, respectively (Figure 5C). On the other hand, PD98059 decreased the activation of ERK both in cells treated with FN or LAM (Figure 5B and 5D) and increased the activation of Akt about 2.7-fold and 2.1-fold compared to FN and LAM treated controls, respectively (Figure 5D). Treatment with both LY294002 and PD98059 followed by treatment with FN or LAM decreased activation of both Akt and ERK (Figure 6A, B and 6C). These data suggest cross-talk between MEK-ERK and PI3K-Akt pathways in FN and LAM-induced enhancement of IPC differentiation. We then analyzed the effects of treatment with LY294002 and PD98059 on the expression of insulin and Glut2. Quantitative RT-PCR showed insulin and Glut2 expression increased by treatment with PD98059 or LY294002 (Figure 5E and 5F). However, treated with both LY294002 and PD98059 decreased the expression of insulin and Glut2 (Figure 6D). Using the vector-based RNAi approach, we further showed knocking down Akt or ERK failed to abrogate FN or LAM-induced enhancement of IPC differentiation. Only knocking down both of Akt and ERK inhibited the enhancement of IPC differentiation by adding ECM (Figure 7). These results all together point out FN and LAM both increased IPC differentiation by Akt and ERK activation.

## Discussion

There is a widespread interest in finding alternative sources of β-cells for tissue replacement strategies in diabetes. MSCs have been used for cell-based therapy in regenerative medicine and tissue engineering. In the current study, we demonstrate adding ECM does not influence the expression of the neural precursor marker and islet transcription factors, but heightens IPC differentiation. Because MSCs spontaneously express the neural precursor maker, Nestin, and transcription factors of the endocrine pancreas developmental pathway such as Nkx6.1 and Ngn3 [8,10], expression of these markers or factors is insufficient to trigger IPC differentiation, which requires further pathways to complete.

ECM provide a dynamic microenvironment to regulate cell morphology, motility, gene expression and survival of adherent cells [17]. FN and LAM constitute the major ECM of pancreas islet cells. Laminin-1 has been reported to promote the differentiation of fetal mouse pancreatic β-cells [18]. Both FN and LAM have been shown to affect the proliferation and insulin release of β-cell [15,19,20]. The current study demonstrates treatment of MSCs with FN or LAM enhances IPC differentiation with increases in insulin and Glut2 gene expressions, proinsulin and insulin protein levels, accumulation of cytoplasmic granules including α, β, and δ granules, and insulin release in response to elevated glucose concentration. Failure to form typical β-cell granules was noted in the IPCs derived from embryonic stem cells [21]. Thus, the appearance of three-kinds of pancreas cytoplasmic granules in these results further indicates the complete achievement of IPC differentiation by adding FN or LAM during differentiation.

Although the benefits of ECM on insulin expression and release are known in β-islet cells [15,19,20], the underlying mechanisms are not clear. This paper demonstrates ECM such as FN or LAM enhances IPC differentiation by activating Akt and ERK. The ERK pathway is the cascade most often associated with signaling mechanisms involved in cell proliferation and cell cycle progression but more recently also in apoptosis [22]. Akt protein, a serine/threonine kinase promotes cell cycle progression, cell survival, and tumour cell invasion [23]. The β-cell proliferation and differentiation are regulated by various growth factors and hormones, including insulin-like growth factor 1 (IGF-1). Treatment of islets with IGF-1 induces GRF-1-dependent activation of downstream signals such as Akt and ERK to maintain a normal β-cell number and function [24]. However, there are few, if any, papers reporting the involvement of ERK or Akt in ECM or biomaterials-induced enhancement of IPC differentiation.

Although, when bone marrow-derived endothelial cells [25] or stem cells [26] were transplanted, they migrated to the site of pancreatic β-cell injury and initiated pancreatic regeneration. These data suggest the paracrine effects of bone marrow cells on supporting endogenous cells to regenerate. However, the potential of bone marrow stem cells or MSCs to differentiate into IPCs has been demonstrated in vitro and in vivo. Bone marrow cells when transplanted into lethally irradiated recipient mice expressed insulin in the pancreatic islets of the recipient mice [27]. This study and others [8] have demonstrated human MSCs spontaneously expressed transcription factors of the endocrine pancreas developmental pathway. It has also been shown mouse marrow MSCs cultured in high glucose for 4 months or rat marrow MSCs cultured with pancreatic extract induces several β-cell-specific genes [28]. Here, adding FN or LAM in pellet suspension culture efficiently induce IPC differentiation by MSCs and succeeded in developing glucose responsive IPCs in vitro. Therefore, the current study offers a potential approach to generate IPCs from MSCs by adding ECM or biomaterials in pellet suspension culture, and demonstrates IPC differentiation by MSCs may be modified in the future by controlling Akt and ERK signaling pathways involved in ECM interaction.
